# Performance of malaria rapid diagnostic test in febrile under-five children at Oni Memorial Children’s Hospital in Ibadan, Nigeria, 2016

**DOI:** 10.11604/pamj.2018.30.242.13268

**Published:** 2018-08-01

**Authors:** Nurudeen Ayobami Adebisi, Hannah Odunola Dada-Adegbola, Magbagbeola David Dairo, IkeOluwapo Oyeneye Ajayi, Olufemi Olamide Ajumobi8

**Affiliations:** 1 Nigeria Field Epidemiology and Laboratory Training Program, Abuja, Nigeria; 2 Department of Medical Microbiology and Parasitology, College of Medicine, University of Ibadan, Nigeria; 3 Department of Epidemiology and Medical Statistics, College of Medicine, University of Ibadan, Nigeria; 4 African Field Epidemiology Network, Abuja, Nigeria; 5 National Malaria Elimination Programme, Federal Ministry of Health, Abuja, Nigeria

**Keywords:** Malaria, rapid diagnostic test, microscopy, parasitemia, sensitivity, specificity

## Abstract

**Introduction:**

The World Health Organization (WHO) recommends testing of suspected malaria cases before treatment. Malaria rapid diagnostic test (mRDT) has been recommended for this purpose in endemic countries where microscopy is not accessible. However, its diagnostic performance remains a concern in clinical settings. We assessed diagnostic performance of RDT among febrile under-five children (U5) presenting at Oni Memorial Children's Hospital, Ibadan (OMCH).

**Methods:**

A cross-sectional study was conducted among 370 febrile U5 attending OMCH February to May, 2016. We examined their finger prick blood samples for malaria parasitaemia using CareStart^TM^ histidine rich protein II (HRP-2) RDT and microscopy. The sensitivity, specificity, positive and negative predictive values (PPV, NPV), false positive (FP), invalid rates (IR), likelihood ratio of positive and negative tests (LRP and LRN), were calculated.

**Results:**

Mean age of the children was 28.17 ± 15.59 months. Malaria prevalence was 21.6% and 15.1% by mRDT and microscopy, respectively. Sensitivity of CareStart^TM^ HRP-2 RDT was 94.6% (95% confidence interval (CI): 84.2-98.6), specificity: 91.4% (CI: 87.6-94.2), PPV: 66.3% (CI: 54.7-76.2), NPV: 98.9% (CI: 96.8-99.7), FPR 6.5%, IR 8.1%, LRP:10.6 and LRN:0.1.

**Conclusion:**

Diagnostic performance of CareStart^TM^ used in the study met the ≥ 95% sensitivity at 100 parasites/µL recommended by WHO. This finding provides clinical evidence that testing before anti-malarial treatment as recommended by WHO will identify cases of malaria infection and reduce unnecessary use of drugs. Healthcare workers should be educated on diagnostic accuracy of mRDT and adhere to the WHO's test-treat strategy for anti-malaria therapy.

## Introduction

Malaria is a life threatening tropical disease responsible for significant morbidity and mortality especially, among children and pregnant women, with 48 to 304 million new cases of malaria infections reported annually worldwide. Nigeria accounted for up to 29% of the total estimated malaria cases and 26% of deaths in the African region [1]. Accurate diagnosis of malaria still remains a challenge because of lack of pathognomonic symptoms, as fever which is one of the commonest symptoms of the disease in children has numerous causes. Infection with bacteria, viruses, protozoa or fungi can manifest as febrile illnesses, thereby presenting with common overlapping manifestations making clinical diagnosis very difficult and leading to undue delay in initiating treatment of fever in children, with the attendant complications including deaths [2]. In the tropics and malaria-endemic regions, most fevers are presumed to be due to malaria and are treated empirically as such [3]. There is mounting evidence that a significant proportion of these febrile illnesses are non malaria [4, 5]. These non-malarial febrile illnesses have been defined as infectious diseases in patients who present with undifferentiated fever and require malaria rapid diagnostic tests or microscopy, but in whom these tests were negative [6]. Despite the awareness of non malaria causes of febrile illness, empirical treatment of fevers, with antimalarial medicines continues in resource-poor settings. In order to limit the development of resistance to drugs and mitigate the consequences of failure of therapy, the World Health Organization advocate for test-based management of malaria and restricting artemisinin-based combination therapy (ACT) to only parasitologically confirmed cases [7]. Diagnosis of malaria based on blood film microscopy using thick and thin smears stained with Giemsa has remained the gold standard for many years [8, 9]. However, the inherent limitations of microscopy such as non-availability of technical expertise and erratic electricity supply and dependence on sophisticated equipment have severely hindered its universal routine use particularly in a busy outpatient clinic and rural settings [10].

This necessitated the introduction of mRDT kits in the early 1990s with the potential to overcome the weaknesses associated with microscopy [11]. Malaria rapid diagnostic tests are based on malaria antigen detection and are meant to differentiate malaria from non-malarial febrile illness, with a turnaround time of less than thirty minutes and have been shown to be effective [12]. CareStart^TM^ malaria an HRP2-based immunochromatographic test which detects HRP2 antigen that is specific for *Plasmodium falciparum* has a very high performance level as reported by the most recent mRDT evaluation programme [13] and is among the mRDTs approved for procurement by National Malaria Elimination Programme (NMEP). Nigeria adopted mRDT as a diagnostic tool, where microscopic diagnosis is not feasible [14], however, the diagnostic performance of the mRDT and its routine usage has remained a source of concern to health-care providers in clinical settings [15, 16]. Some of the challenges include erratic supply, non-availability of test kits and health care worker's perspectives about its accuracy and reliability with resultant non-adherence to test result when used [15, 16]. This may not be totally unconnected with varying reports of sensitivity of different types of mRDT kits ranging from 94.3% in Ibadan [17], 47.0% in Port Harcourt [18], 40.3% in Zamfara [19] to as low as 8.3% in Maiduguri [20]. The level of sensitivity of the different mRDT kits reported in majority of the studies fall far below the recommended level of ≥ 95% sensitivity by WHO. Therefore, the poor use of CareStart^TM^ RDT despite availability at a secondary level hospital, Oni Memorial Children's Hospital Ibadan (OMCH) could largely be as a result of varying reports of sensitivity in Nigeria. We therefore assessed the diagnostic performance of CareStart^TM^ RDT among febrile under-five children (U5) who presented at OMCH.

## Methods

**Study area**: A hospital-based cross-sectional study carried out at Oni Memorial Children's Hospital, Ibadan in Ibadan South West Local Government area, Nigeria from February to May, 2016. Oni Memorial Children's Hospital was purposively selected because it is the only secondary level health facility dedicated to children below 10 years in Oyo State. The hospital is a 65-bedded capacity facility, consists of an emergency unit, intensive care, out-patient unit and 2 specialised units for Heamatology Day Care and Neurological cases. It serves as a referral center for all the health centers within and outside Ibadan. It also provides routine immunization and child welfare services. An average of 900 children of which 500 (55.6%) are under-five are attended to on a monthly basis, while about 750 (83%) of these children are presumptively diagnosed as malaria cases thereby necessitating laboratory confirmation. Though, CareStart^TM^ RDT was available at the OMCH, microscopy remained the mainstay of diagnosing malaria.

**Sample size estimation**: The number of participants for the study was calculated using the following formula:

$$\text{n}=\frac{{\left({\text{z}}_{\alpha }\right)}^{\text{z}}\text{Pq}}{{\text{d}}^{2}}$$

[21] where: n = minimum number of participants needed; Z_α_ = standard normal deviate corresponding to 2-sided level of significance at 5% = 1.96; p = prevalence of malaria parasitaemia among under-five children presenting with malarial at a general hospital in Ota, Ogun State, 70% [22]. q = 1 - p; d = level of precision at 5%

$$\text{n}=\frac{{\left({\text{z}}_{\alpha }\right)}^{\text{z}}\text{Pq}}{{\text{d}}^{2}}$$

(1.96)^2^ x 0.70 x 0.30/(0.05)^2^ = 323 Adjusting for non-response rate (10%) calculated using the following formula:

= n x 1/(1-r) Where: n = sample size calculated; r = non response rate (10%); = 323 x 1/(1-0.1) = 359. The minimum sample size for the study was 370.

**Recruitment of participants and data collection:** A total of 370 febrile under-five children who presented at the emergency and general out-patient (GOP) units of the OMCH with history of fever (axillary temperature > 37.5°C) and a provisional diagnosis of malaria and whose parent/caregivers gave informed consent for the study were recruited consecutively over a period of two months. We excluded all children who were severely ill and because we could not analyze the drug level of those who have taken any anti-malaria drug, they were also excluded. Data on demographic characteristics, child characteristics and episodes of malaria illness were collected from the caregivers using pre-tested interviewer administered questionnaire.

**Laboratory examination:** Capillary blood sample of each febrile child obtained by finger prick was collected on frosted end slides and CareStart^TM^ Histidine-rich protein 2 (HRP-2) RDT Cassette. Thick and thin blood films were prepared, stained with Giemsa same day and examined microscopically according to WHO standards [23]. Parasite density per microliter of blood (parasitaemia) was determined according to the number of parasites per 200 white blood cells (WBC), assuming a total WBC count of 8,000 /μL and expressed as follows: Parasite density µL^-1^ = parasite count/number of white blood cells counted × 8000 Microscopic examination of all stained slides was conducted by a trained WHO certified malaria microscopist with 7 years of experience, following standard methods [23]. Each sample was tested with CareStart^TM^ malaria Pf RDT, Lot No. M015C01 manufactured in March, 2015 by Access Bio Incorporation, New Jersey, USA. This was obtained directly from the pharmacy Unit of OMCH through United State agency for International Development (USAID) representative. All the participants were tested by adhering strictly to manufacturer's guidelines.

**Quality control**: The positive control check of the RDT was done with positive (dilutions at 200 and 2000 parasite/µL of blood) and negative (zero parasitaemia) confirmed by microscopy.

**Data analysis**: Data was analyzed using Statistical Package for Social Sciences (SPSS) software version 16.0. Data were summarized using mean, frequencies, proportions and 95% confidence interval (CI). Parameters for mRDT diagnostic performance was calculated using the standard WHO format as follows: (a) True positive (TP) = sample is positive by both microscopy and CareStart^TM^ HRP-2; (b) True negative (TN) = sample is negative by both microscopy and CareStart^TM^ HRP-2; (c) False positive (FP) = sample is positive by CareStart^TM^ HRP-2 but negative by microscopy; (d) False negative (FN) = sample is negative by CareStart^TM^ HRP-2 but positive by microscopy.

**Ethical consideration**: Ethical approval for the study was obtained from Oyo State Ethical review committee (reference number: AD13/479/144, date: 13/01/2016). Informed consent was also obtained from the caregivers.

## Results

Overall, 370 febrile children were included in the analysis. The mean age of the children was 28.2 months (standard deviation: 15.6). The male participants constitute 211 (57.0%), other children characteristics are shown in Table 1. The most common presenting complaint other than fever was loss of appetite (23.2%), cough and catarrh (22.4%) as well as body weakness (15.4%). While all the participants presented with history of fever, only 123 (33.2%) was febrile (temperature ≥ 37.5°C) at time of presentation (Table 1). The prevalence of malaria or the proportion of positive cases among the febrile U5 were 21.6% (80/370) and 15% (56/370) by mRDT and microscopy, respectively (Table 2). The CareStart^TM^ HRP-2 detected 53 true positives (TP), 27 false positives (FP), 287 true negatives (TN) and 3 false negatives (FN). The microscopy detected 56 malaria positive cases among the participants, 55 *Plasmodium falciparum* and 1 *Plasmodium malariae*, while the RDT detect 80 positive cases of *Plasmodium falciparum* (Table 2). The Sensitivity of CareStart^TM^ HRP-2 RDT was 94.6% (95% CI: 84.2-98.6) and specificity of 91.4% (CI: 87.6-94.2) (Table 3).

**Table 1:** Clinical presentation, fever duration and care seeking for children under five with history of fever at OMCH, Oyo State, February to May, 2016 (N = 370)

Characteristics	Frequency (%)	
Symptoms at presentation	Body weakness	57 (15.4)
	Abdominal pains	19 (5.1)
	Vomiting	45 (12.2)
	Loss of appetite	86 (23.2)
	Cough and catarrh	83 (22.4)
	Diarrhoea	45 (12.2)
	Headache	35 (9.5)
Child received medicine since symptom onset	Yes	355 (95.9)
	No	15 (4.1)
Medication given to the child	Antimalaria	199 (53.8)
	Antibiotics	17 ( 4.6)
	Paracetamol	135 (36.5)
	Herbs	19 (5.1)
Duration of health care seeking	Same day of noticing fever	24 (6.5)
	Next day after fever onset	104 (28.1)
	≥ 2days after fever onset	242 (65.4)

**Table 2:** Distribution of cases of malaria detected by parasitological technique in children with history of fever at OMCH, Oyo State, February to May, 2016

Malaria parasitaemia			
	Microscopy Positive frequency (%)	Negative frequency (%)	Total frequency (%)
**RDT positive**	53 (94.6)	27 (8.6)	80 (21.6)
**RDT negative**	3 (5.4)	287 (91.4)	290 (78.4)
**Total**	56 (100.0)	314 (100.0)	370 (100.0)

**Table 3:** Diagnostic performance of CareStartTM HRP-2 RDT among children with history of fever

Characteristics	Percentage (%)	95%(confidence interval)
Sensitivity	94.6%	84.2-98.6
Specificity	91.4%	87.6-94.2
Positive predictive value	66.3%	54.7-76.2
Negative predictive value	98.9%	96.8-99.7
False positive rate	6.5%	-
Invalid rate	8.1%	-
Likelihood positive ratio	10.6	-
Likelihood negative ratio	0.1	-

## Discussion

Despite several interventions, malaria still continues to be a major public health challenge especially among under five children. Malaria parasitaemia prevalence of 15.1% and 21.6% was observed with light microscopy and mRDT respectively in this study. This observed prevalence is lower than previously reported prevalence among children presenting at health facilities in Nigeria which ranged between 27.7% and 84.7% in studies by Ikeh and Teclaire [24], (56.9%), Oladehinde et al [22, 25], (70.0%,84.7%), Okoli and Solomon [26] (51.0%) and Elechi et al [20] (27.7%). The differences in the prevalence rates may be due to some reasons which include, but not limited to, the seasonal variation, location of the study (rural or urban). However, wide spread use of ACTs in the Country, insecticide treated nets (ITN) and other preventive measures may have contributed to the low malaria prevalence reported in our study, which is in line with a declining trend in malaria prevalence in Nigeria [27, 28]. Therefore, febrile U5 children should not be assumed to have malaria without testing. While, testing before antimalarial will reduce the abuse or over prescription of antimalarial drugs and will allow early diagnosis of other cause of febrile illness. CareStart^TM^ RDT HRP2 Pf shows a good performance with high sensitivity of 94.6% and comparable to the 94.3% and 93.0% sensitivity reported in Ibadan by Falade et al [17] and in Sokoto by Sani et al [29] respectively. However, it varied markedly from sensitivity of 100% ,78.4%, 69.6%, 42.31% and 40.3% reported in Kaduna by Ajumobi et al [30], Jos by Sheyin and Bigwan [31], Lagos by Ben-Edet et al., [32], Enugu by Oguonu and Okafor [33], and Zamfara by Abdulkadri et al [19], respectively.

The disparity in reported sensitivity may not be because the studies used different kits, for instance while Ajumobi, Falade, and Jeremiah all used SD Bioline malaria Pf RDT, they still reported discordant sensitivity of 100%, 94.3% and 47% respectively. Also for the CareStart^TM^ RDT HRP2 Pf, while the current study gave a sensitivity of 94.6%, Sheyin and Bigwan, and Abdulkadri reported 78.4% and 40.3% respectively. These observed conflicting reports may be due to the functionality of RDTs which remains unable to detect parasites at low densities (< 200-400/ µL). However, other factors such as storage which were not considered in the current study may be responsible for the differences which has eroded the confidence of end users. Although the RDT specificity was high (91.4%), 27 children still had false positive results while microscopy was negative. This may imply that the children did not have active malaria, or had incomplete treatment with antimalarial drug, however, the positive RDT result could be attributed to delayed clearance of antigen from the circulation. It has been shown that malaria antigen clearance could be delayed for up to 4 weeks even, when treatment was successful Abdulkadri et al [19]. High specificity of RDT kits will reduce unnecessary treatment with antimalarial drug and improve early diagnosis of other causes of febrile illnesses in all settings. Furthermore, the three observed children with false negative result may suggest that the children were infected with Plasmodium species other than falciparum or caused by other non-HRP2 containing P. falciparum. The current finding of 98.9% negative predictive value of CareStart^TM^ also support the validity on its use in excluding/eliminating malaria as the cause of febrile illness and allows early decision making in looking out for other causes of febrile illness in the under-fives, which will enhance their survival, thereby reducing mortality in under-fives. The other findings which include invalid rate of 8.1% are within the acceptable rates according to WHO assessment of RDTs 2014-2015 round six [13].

## Conclusion

The diagnostic performance of CareStart^TM^ HRP-2 RDT met the ≥ 95% sensitivity at 100 parasites/µL recommended by WHO. The study provides the clinical evidence to justify its use for testing before treatment as recommended by WHO. Use of RDT should be encouraged for diagnosis of malaria using a protocol that encourage further evaluation of febrile children with negative RDT results using microscopy. Efforts should be made to educate healthcare workers on the importance of RDT test kits and enforcement of protocols that supports the WHO's test-treat strategy for anti-malaria therapy. There should be regular communication of availability of mRDTs among all stakeholders in every health care facilities and also Government at both federal, state and local levels should limit the number of mRDT product types for the country based on local data on performance. Also the varying sensitivities in different parts of Nigeria calls for more study to unravel the circumstances affecting the performance of the rapid test kits.

**Limitations of the study:** Report on the use of antimalarial drug before presentation cannot be verified but we elicited this from the caregivers as much as possible. The negative results could not be confirmed by polymerase chain reaction (PCR) because of limited funds.

### What is known about this topic

- Accurate diagnosis of malaria remains a challenge in Nigeria and empirical treatment of fevers, with antimalarial medicines continues in resource-poor settings;
- Diagnosis of malaria based on blood film microscopy using thick and thin smears stained with Giemsa has remained the gold standard for many years;
- Nigeria adopted mRDT as a diagnostic tool, where microscopic diagnosis is not feasible but there have been varying reports of sensitivity of different types of mRDT kits ranging from 8.0 % to 94.3% in Nigeria.

### What this study adds

- Diagnostic performance of CareStart^TM^ HRP-2 RDT met the ≥ 95% sensitivity at 100 parasites/µL recommended by WHO;
- The study provides the clinical evidence to justify its use for testing before treatment as recommended by WHO;
- Government at both federal, state and local levels should conduct lot testing of batches of mRDT on a regular basis to ensure the mRDT in use at health facilities are of high diagnostic performance.

## Competing interests

The authors declare no competing interest.
